# Predictions of potential geographical distribution of *Diaphorina citri* (Kuwayama) in China under climate change scenarios

**DOI:** 10.1038/s41598-020-66274-5

**Published:** 2020-06-08

**Authors:** Rulin Wang, Hua Yang, Mingtian Wang, Zhe Zhang, Tingting Huang, Gang Wen, Qing Li

**Affiliations:** 1 College of Agronomy, Sichuan Agricultural University, Chengdu, Sichuan 611130 China; 2 Sichuan Provincial Rural Economic Information Center, Chengdu, Sichuan 610072 China; 3 Key Laboratory of Ecological Forestry Engineering of Sichuan Province/College of Forestry, Sichuan Agricultural University, Chengdu, 611130 China; 4 Sichuan Meteorological Observatory, Chengdu, Sichuan 610072 China; 5 Bureau of Agriculture of Yibin City, Yibin, Sichuan 644000 China

**Keywords:** Ecological modelling, Ecological modelling

## Abstract

Climate change significantly affects geographic distribution of plants pests and diseases worldwide. Understanding the influence of future climate change on the suitable areas of *Diaphorina citri* (Kuwayama) in our country and taking timely countermeasures are crucial for improving the effectiveness of control of pest. Based on the occurrence points of *D. citri* and the selected environmental variables, the potential suitable areas of this pest under climate change scenarios in China were predicted by using MaxEnt and GIS tools. Our results showed that the higly suitable area were mainly located in Guangxi, Guangdong, Fujian, Southern Zhejiang, Southern Jiangxi, Eastern Hunan, Southwestern Guizhou, and the area was 43.7 × 10^4^ km^2^. Areas of moderate and low suitability were centered on areas of high suitability and radiate to the North successively, with an area of 59.28 × 10^4^ km^2^ and 93.46 × 10^4^ km^2^ respectively. From current to 2070 s, the areas of the highly suitable areas will increase, and the geometric center of the highly and total suitable areas will move to north under three climate change scenarios.

## Introduction

Climate change has become one of the global hot environmental issues, and agriculture is the most sensitive area affected by climate change^1^. Extreme weather and climate events such as global high temperatures, drought, rainstorms, floods and hail caused by climate change will have an important impact on the growth period, yield, quality of crops, occurrence and development of diseases and pests, thus increasing the instability of agricultural production, changing agricultural production structure and production conditions, and increasing agricultural cost and investment substantially^2^. According to the fifth assessment report of the UN’s intergovernmental panel on climate change, the global average surface temperature has increased by 0.85 °C in the past 130 years and 1.45 °C in Asia from 1901 to 2015^3^. In the past 30 years, surface temperatures per 10 years have been higher than any 10 years since human records, and the highest temperatures have been recorded in more than a decade since 2000^4^. In the context of global warming, the annual average surface temperature in China has increased by 1.1 °C in the past 50 years, which is significantly higher than the average temperature increase rate in the same period in the global or northern hemisphere^5^.

It has been confirmed that the reproduction and development of agricultural diseases and pests are affected by biological and abiotic factors^6^. Meteorological factor is one of the environmental factors closely related to the distribution, occurrence and development of pests and diseases. Global climate change has profoundly changed the composition, structure, function and succession of insect community in agroforestry ecosystem, expanded insect distribution area, increased generation and ecological adaptability variation, and then affected the internal relationship among plants, pests and natural enemies, and led to the outbreak of some pests^7^. Climate change has affected the growth and development, metabolic rate, survival and propagation, migration and diffusion of pests, and increased the probability of pest diffusion from low latitude to high latitude^8^.

The Asian citrus Psylla, *Diaphorina citri* Kuwayama (Hemiptera: Psyllidae), which feeds on almost all citrus varieties, is the main pest in the new shoot stage of host plants^9^. It ingests phloem with the piercing-sucking mouthparts, which causes the young shoots to wither, the new shoots to bend, and the young leaves to deform and twist. White excreta of nymphs can induce sooty mildew on branches and leaves and affect photosynthesis of plants^10^. However, compared with the direct feeding damage, the greatest harm of *D. citri* is the transmission of the pathogen causing citrus huanglongbing (HLB)^11,12^. Citrus HLB, a phloem-limited bacteria disease, is a most destructive and devastating disease of citrus in Asia, Africa, North and South America^13^. All commercially cultivated citrus is susceptible and varieties tolerant to the disease are not yet available. It causes substantial economic losses by reducing fruit production, shortening the lifespan of the tree, but no effective cure has been found^14^. As of 2018, China’s citrus cultivation area is about 2.6 million hectares, which is the world’s largest citrus producing country^15^. According to statistics, HLB disease occurs in 17 major citrus producing provinces in China, with an area of more than 200000 hectares, causing great economic losses every year^16,17^. In recent years, with global warming, the northern boundary of citrus HLB and *D. citri* in China has shifted significantly northward^18,19^.Considering the uniqueness of *D. citri* to transmit citrus HLB, it is necessary to study the impact of climate change on the geographical distribution of *D. citri*.

Prediction of potential geographic distribution of pests and diseases is one of the important research components of quantitative risk assessment of pests^20^. MaxEnt model is non-commercial software with high accuracy and small sample demand. It has been widely used in the fields of ecology, species evolution, biosafety and conservation biology since it came out in 2004^21^. Hu *et al*. used MaxEnt to project the distribution of the overwintering population of *Sogatella furcifera*, in Yunnan, China^22^. Srivastava *et al*. assessed the potential distribution of Asian Gypsy Moth in Canada using MaxEnt and GARP^23^. Kumar *et al*. used MaxEnt to generate a district-level map of the potential risk of invasion by *Phenacoccus solenopsis* Tinsley in India^24^. All the above studies have shown that MaxEnt model can provide scientific basis for assessing the possibility of pest invasion, spread and colonization, and formulating corresponding prevention and control strategies.

For *D. citri*, researchers world-wide have studied its biological and ecological characteristics^25,26^, the transmission of the pathogen causing HLB^27,28^, the principle of host selection^29^ and comprehensive control measures^30,31^, but there are not many special studies on its geographical distribution in China. In 2015, Wang *et al*. applied the CLIMEX model to analyze the potential habitats and the spreading trend of *D. citri* in China^32^. Studies have shown that different models can effectively verify and supplement previous studies. In view of this, MaxEnt model with higher accuracy was selected to simulate the distribution pattern and migration rule of *D. citri* in China under different emission scenarios. The relevant research results of this paper will provide theory and data support for the early warning, quarantine inspection and comprehensive prevention and control of *D. citri*.

## Results

### Selection of the key environmental factors

**Figure 1:**
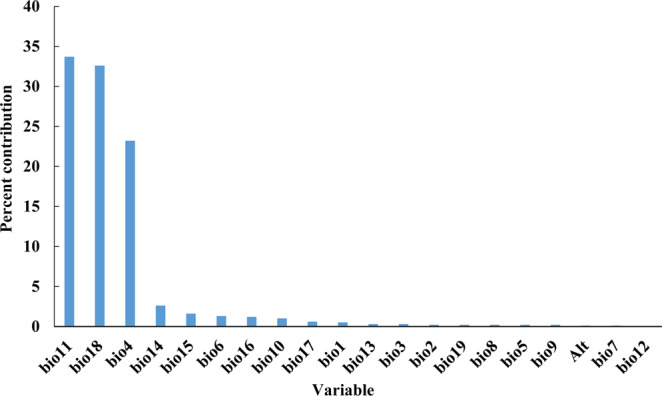
Percent contribution of the environmental variables to the MaxEnt model.

MaxEnt is a mathematical model based on the principle of climate similarity; it is used to explore the correlation between geographical distribution and climatic factors. A choice of climatic factors is the key to determining the accuracy of the simulation. Therefore, referring to the method in Methods section, we screened the environmental factor**s** used ArcGISental variables to build the model. According to the introduction of the method part, four extreme temperature variables (bio5, bio6, bio10 and bio11) were reserved as necessary variables. Then to extract the attribute values of the environmental variables corresponding to 135 distribution points, and use SPSS to calculate the Pearson correlation coefficient between variables (Table S1). We calculated the importance of each variable to the modeling using the knife cutting method (Fig. 1). By comparing the biological characteristics and correlation coefficient, 9 variables were reserved for modeling, which were bio4, bio5, bio6, bio10, bio11, bio14, bio15, bio16, and bio18.

### Model performance

**Figure 2:**
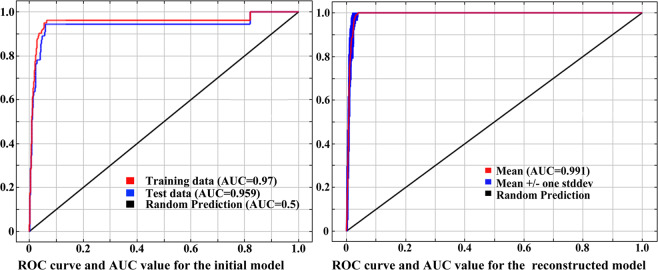
ROC curve and AUC value for the initial model and the reconstructed model under current situation.

**Figure 3:**
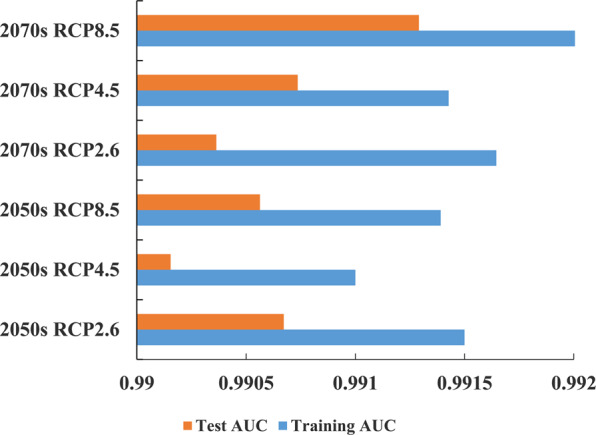
ROC curves and AUC values for the final models in the future.

Figure 2 shows the ROC curve of the initial model and the reconstruction model under current climate situation. The AUC values of the training data and the test data of the initial model are 0.97 and 0.959, respectively, and the mean AUC value of 10 replicates of the reconstruction model was 0.991 (Fig. 2). Figure 3 shows the AUC values in the future. The results showed that the mean AUC values of training data were all greater than 0.99. According to the evaluation criteria, the accuracy of the two models was ‘excellent’. The above results proved that the model can be used to study the potential distribution simulation of *D. citri* in China.

### The potential distribution of *D. citri* in China under current climate condition

**Figure 4:**
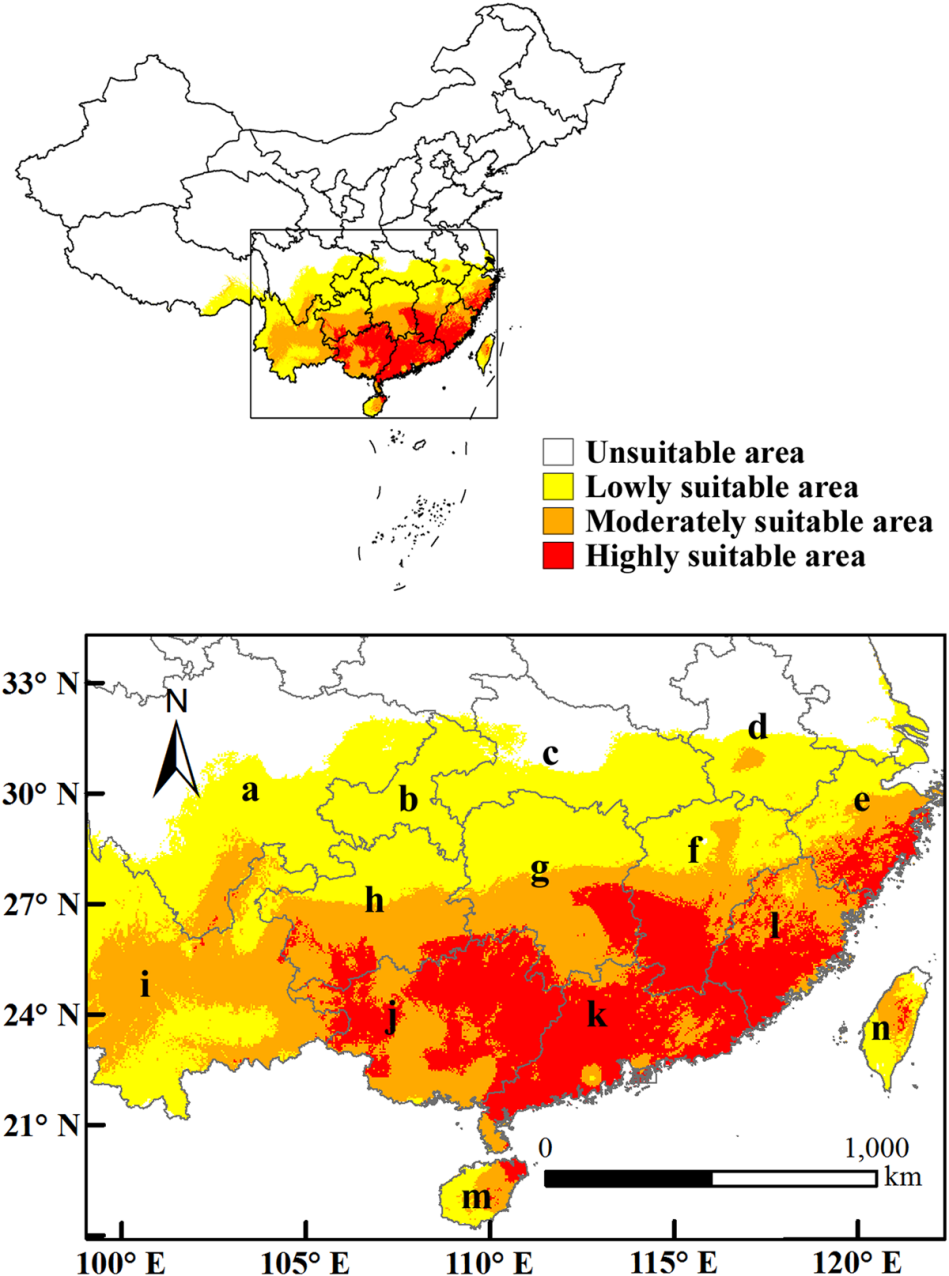
Potential suitable distribution of D. citri in China under current situation. In the figure, a (Sichuan), b (Chongqing), c (Hubei), d (Anhui), e (Zhejiang), f (Jiangxi), g (Hunan), h (Guizhou), i (Yunnan), j (Guangxi), k (Guangdong), l (Fujian), m (Hainan), n (Taiwan).

The results (Fig. 4) showed that under current climate condition, the highly suitable area of *D. citri* were mainly located in Guangxi, Guangdong, Fujian, Southern Zhejiang, Southern Jiangxi, Eastern Hunan, Southwestern Guizhou, with an area of 43.7 × 10^4^ km^2^, which occupied 4.55% of the land surface of China. The moderately and lowly suitable areas were centered on the highly suitable area and radiate to the North successively, with an area of 59.28 × 10^4^ km^2^ and 93.46 × 10^4^ km^2^ respectively. The moderately suitable areas were distributed in Yunnan, Southern Guizhou, Southern Hunan, central Jiangxi, Northern Fujian, central Zhejiang, Eastern Hainan, central Taiwan and Southeastern Sichuan, which were more widespread than the highly suitable area. The area of the total suitable area composed of highly and moderately suitable areas was 102.98 × 10^4^ km^2^, that made up for 10.73% of its land mass.

### Potential distribution of *D. citri* in China under climate change scenarios

**Figure 5:**
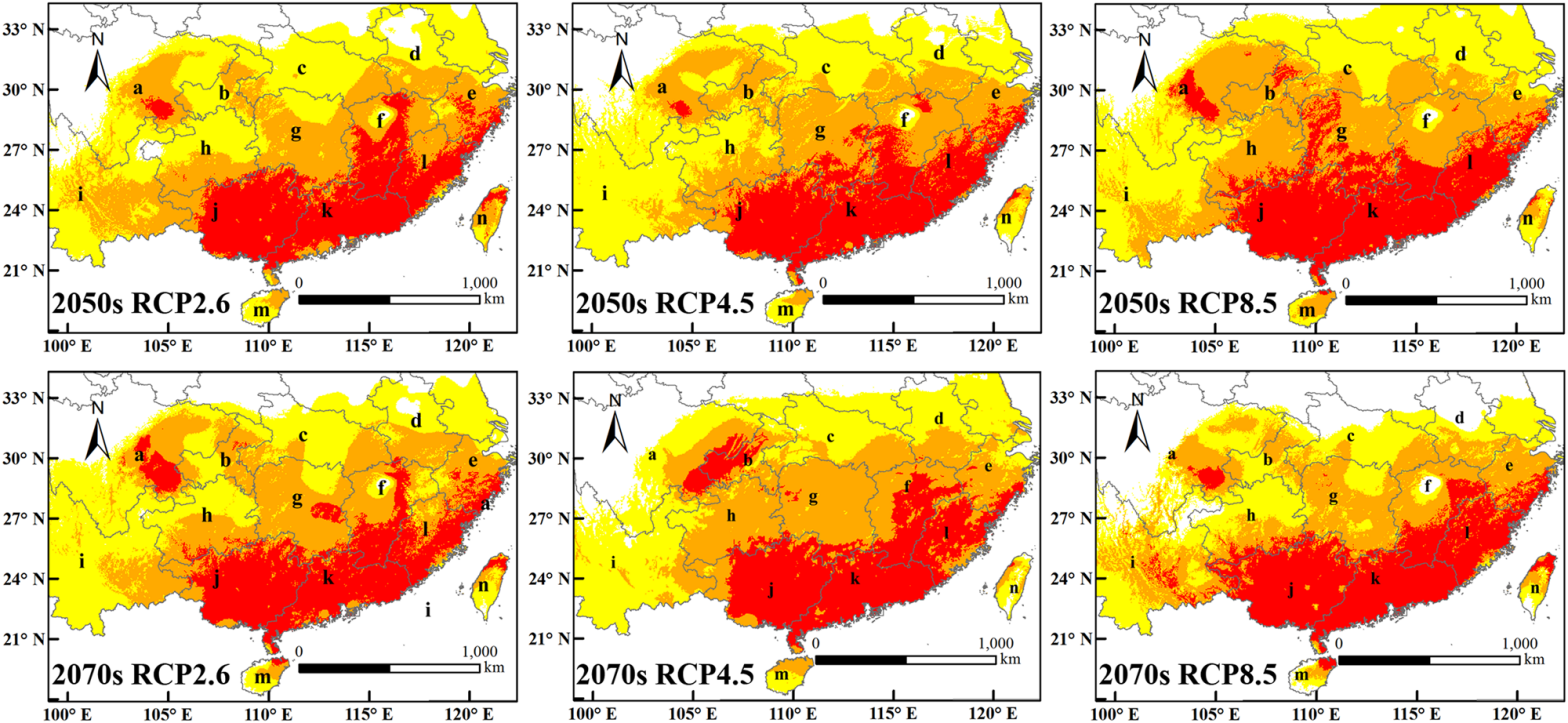
Potential suitable distribution of *D. citri* under climate change scenarios.

Under the three climate change scenarios, the areas of the highly suitable areas will increase gradually (Fig. 5). By 2050 s, the areas increased to 49.61 × 10^4^ km^2^ (RCP2.6), 51.71 × 10^4^ km^2^ (RCP4.5) and 50.08 × 10^4^ km^2^ (RCP8.5), while by 2070 s, the areas will increased to 55.27 × 10^4^ km^2^ (RCP2.6), 59.26 × 10^4^ km^2^ (RCP4.5) and 57.01 × 10^4^ km^2^ (RCP8.5).

In the future, the change trend of the areas of the moderately suitable areas is similar to that of the highly suitable areas, which shows that the area will increase significantly. By 2050 s, the areas will be increased to 72.09 × 10^4^ km^2^ (RCP2.6), 73.97 × 10^4^ km^2^ (RCP4.5) and 71.35 × 10^4^ km^2^ (RCP8.5), while by 2070 s, the areas will be increased to 76.92 × 10^4^ km^2^ (RCP2.6), 82.18 × 10^4^ km^2^ (RCP4.5) and 65.28 × 10^4^ km^2^ (RCP8.5) (Fig. 5).

### The trajectory of the geometric center of *D. citri* in the future

In order to analyze the influence of climate change on the geographical distribution of *D. citri*, this paper used the spatial analysis function of ArcGIS to calculate the position of geometric center of *D. citri* under different scenarios in China, and calculated the migration distance and direction. The results were as follows.

**Figure 6:**
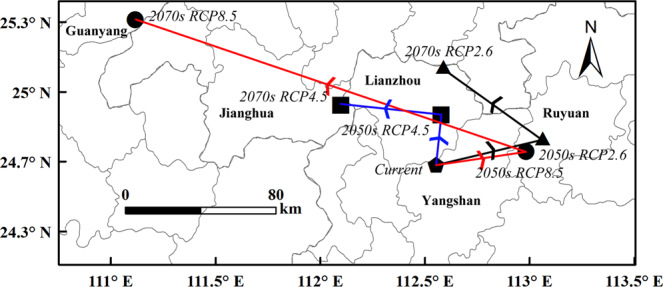
The geometric center of the highly suitable areas during different periods.

**Figure 7:**
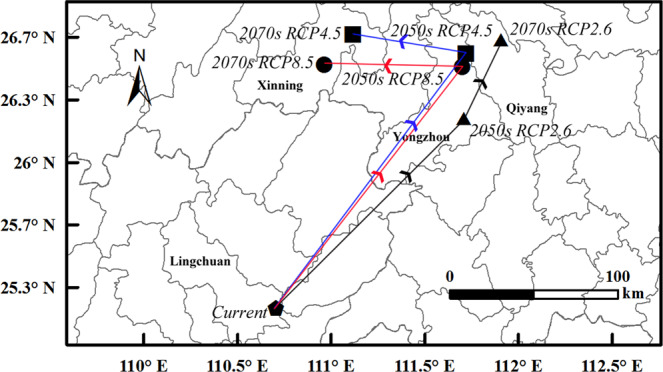
The geometric center of the total suitable areas during different periods.

Under RCP2.6, the geometric center of *D. citri* of the highly suitable areas will move 52.43 km from Yangshan (Current) to northeast to Ruyuan (2050 s), then 58.54 km to northwest to Lianzhou (2070 s). By 2070 s, the center will generally displaced 46.84 km to the northeast (Fig. 6). The geometric center of *D. citri* of the total suitable areas will move 142.57 km from Lingchuan (Current) to northeast to Yongzhou (2050 s), then 46.28 km to northeast to Qiyang (2070 s). By 2070 s, the center will generally displaced 186.82 km to northeast (Fig. 7).

Under RCP4.5, first the center will move 23.84 km from Yangshan (Current) to northeast to Lianzhou (2050 s), then 48.15 km to northwest to Jianghua (2070 s). By 2070 s, the center will generally displaced 53.66 km to northwest (Fig. 6). The geometric center ot the total suitable areas will move 168.34 km from Lingchuan (Current) to northeast to Yongzhou (2050 s), and then 59.19 km to Northwest to Xinning (2070 s). Compare the position of the current and 2070 s, the geometry center will generally move 151.6 km to northeast (Fig. 7).

Under RCP8.5, the geometric center of the highly suitable areas will move 43.47 km from Yangshan (Current) to northeast to Ruanyuan (2050 s), then 271.14 km to northwest (Guanyang, 2070 s). By 2070 s, the center will generally displaced 248.64 km to northwest (Fig. 6). The center of the total suitable areas will move 162.67 km from Lingchuan (Current) to northeast to Yongzhou (2050 s), and then 73.76 km to northwest to Xinning (2070 s), and generally moved 132.24 km to northeast (Fig. 7).

## Discussion

Under the premise of niche conservation, using the local distribution data of a species to analyze the geographical distribution of the species has high accuracy^33^. However, the accuracy of projecting its calculation results to other regions and predicting the distribution probability in this region is often unstable. So, it is not recommended to use presence data from a distant region to model occurrence in another zone^34^. In this paper, we only used the distribution data of *D. citri* in China, which can effectively avoid the above problems.

In this paper, MaxEnt model was used to study the suitable areas of *D. citri* in China under current and future climatic conditions. ArcGIS was used to quantitatively analyze the change of suitable areas and the trajectory of the geometric center in 2050 s and 2070 s. ROC curve was used to evaluate the prediction accuracy of the MaxEnt model, and the results showed that the simulation effect is good, and the results can be used in this study. Nine environmental variables were selected as the key factors, which indicated that temperature played a key role in the distribution of *D. citri*. Previous studies have shown that, low temperature in winter was the main factor limiting population growth, geographical distribution and potential transmission of *D. citri*^35–38^. Therefore, the environmental variables that we selected have certain biological significance, which ensures the accuracy of the simulation results.

We simulated the geographical distribution of *D. citri* under current climate conditions, and the results showed that the area of the total suitable area was 102.98 × 10^4^ km^2^, that made up for 10.73% of its land mass, which was inconsistent with our previous research^39^. This was because in this paper we improved the method of variable selection and updated the species distribution data. Wang *et al*.^32^ studied the past, current and future potential distributions of *D. citri* in China using CLIMEX sofware, and the predicted highly suitable areas were basically consistent with our simulation. However, the results of this study showed that the distribution and area of the moderately area were wider, larger and more northerly, which may be caused by different sample data, environmental variables and simulation theories. According to previous articles^19^, no *D. citri* has been found in Chongqing, Hubei, Anhui and Jiangsu, but this study points out that there were lowly suitable areas for the pest in these provinces. We inferred from this that *D. citri* may continue to spread. Therefore, the agricultural sector should take appropriate quarantine measures to prevent the introduction of *D. citri* to areas with suitable host and climatic conditions.

Studies have shown that the occurrence and prevalence of agricultural pests were closely related to the biological characteristics, host crops, farmland management level and environmental factors^40^. Meteorological and climatic conditions were important environmental factors affecting the occurrence of pests. Climate warming would lead to the increase of the reproduction algebra of pests, the expansion of suitable geographical distribution^41^ and the gradual migration of occurrence boundaries to high latitudes^42^. In recent years, it had been pointed out that with the increasing global warming, the geographical distribution of *D. citri* spreads to the North obviously in China, which also intensified the spread speed and damage scope of citrus HLB^43^. In this study, we predicted the geographical distribution of *D. citri* in China under three climate change scenarios. The results were as follows: From current to 2070 s, the areas of the highly suitable areas firstly decreased and then increased, and the geometric center of the highly and total suitable areas will move to northeast under three climate change scenarios. National Agricultural Technology Extension Center conducted a national survey of *D. citri* in 1982, 2004, 2010 and 2014. The results showed that the northern boundaries of occurrence of *D. citri* in China were 28°45′ N, 29°47′ N, 29°11′ N and 29°29′ N, respectively^44^. Generally speaking, the distribution area of *D. citri* is gradually moving northward. These results confirm that *D. citri* will move northward in the future. Therefore high vigilance should be maintained at the areas designated as unsuitable for *D. citri*. Because of global warming, those areas currently designated as being of lowly suitability or unsuitable may eventually become suitable for *D. citri*.

Under climate change, invasion of new areas by HLB is particularly important. Studies had found that the MaxEnt model uses a complex mechanical learning method, and its transfer ability is low, which affects its application in invasion biology and global change biology^34,45,46^. But these could be compensated for by the use of ensembles of models. In the research of Narouei-Khandan *et al*.^47^ MaxEnt and the Support Vector Machine were selected to draw a consensus map of the global distribution of *D. citri*, which made the prediction result more reliable and provided a more favorable theoretical basis for the effective prevention and control of HLB and *D. citri*. Studies have shown that the ability of active migration and diffusion of *D. citri* was weak. Its geographic diffusion was mainly through the transfer of breeding materials and with the airflow, and was affected by climate, the growth and cultivation conditions of citrus, the situation of orchard medication and the population density of natural enemies of *D. citri*^37,47,48^. Aurambout *et al*.^49^ simulated the potential distribution of *D. citri* in Australia in the future, and also analyzed the impact of climate change on the growth period of host plants. They believed that the increase of global temperature would change the growth frequency of young leaves of citrus, and thus reduce the possibility of invasion and colonization of *D. citri* in Australia. In the next step, we have planned to use ensembles of models such as MaxEnt, GARP and BIOCLIM to further analyze the potential distribution of *D. citri*, HLB, and host plants in China, and integrate their interaction into the model building process effectively. Our results while not incorporating all of the many factors contributing to distribution, are based on climatic change and provide the basis for future refinement and assessment of many of the additional factors.

## Methods

### Environmental variables and Species data

In this study, to analyze the climatic suitability regionalization of *D. citri* in China, we chose climatic factors and altitude factors as initial environmental variables. Climate variables under current situation and altitude data (Table S2), were downloaded from the official website of Worldclim (http://www.worldclim.org/), while variables under climate change scenarios in 2050 s (2041–2060) and 2070 s (2061–2080) were downloaded from the website of CCAFS (http://www.ccafs-climate.org/)^50^ (Table S2).

In the papers of Yang *et al*.^51^ and López-Collado *et al*.^52^, it was pointed out that the geographical distribution of *D. citri* was related to the temperature, especially the extreme low temperature has a significant limiting effect on its distribution. Hall *et al*.^37^ studied the cold hardiness and temperature thresholds for oviposition of the Asian citrus psyllid, and they found that the estimated lower and upper thresholds for oviposition were 16.0 and 41.6 °C, respectively. Kuang *et al*.^53^ analyzed the effects of high temperature on mortality and activity behavior of *D. citri*, and found that the upper limit of survival temperature was 45 °C. Considering the above factors, we selected the extreme temperature as the environmental variable. The selection of the remaining modeling variables was based on the method of Worthington *et al*.^54^.

**Figure 8:**
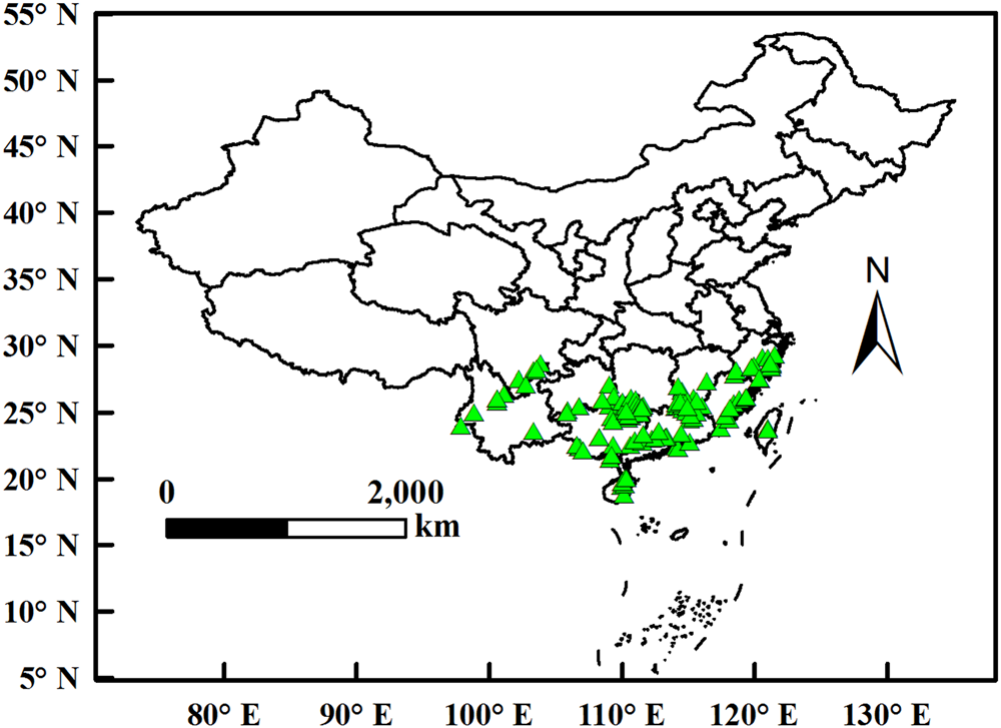
Spatial distribution of occurrence records of *D. citri*.

To obtain the occurrence records of *D. citri* in the world, we accessed two online databases, including the European and Mediterranean Plant Protection Organization (EPPO, https://www.eppo.int/) and the Global Biodiversity Information Facility (GBIF, https://www.gbif.org/)^39^, and consulted many published articles^32,44,51,55,56^. We used Google Earth to proofread the latitude and longitude. In strict accordance with the requirements of MaxEnt, duplicate records, fuzzy records and neighboring records were removed^50,57^. Finally, 135 valid records were retained for constructing the models (Fig. 8). Occurrence records were processed in Microsoft Excel and saved in the format “as.CSV.”^39,50^.

### Modeling method and statistical analysis

MaxEnt, based on the maximum entropy theory, uses species distribution data and environmental data to analyze the distribution of species when maximum entropy occurs. MaxEnt is an ideal tool for studying the geographical distribution of species and has unique advantages^58^, so we selected it as a simulation software for use in this study. MaxEnt software (Version 3.4.1) which is now open source was downloaded from website of the American Museum of Natural History (http://biodiversityinformatics.amnh.org/open_source/maxent/), has excellent predictive performance for plants^50^.

The specific operational steps for MaxEnt are described here. First, we imported the occurrence points of *D. citri* and 19 climatic variables into the MaxEnt software to create the initial model^39,50^. In the initial model, the ‘Random test percentage’ was set as 25, and the ‘Make pictures of predictions’ and ‘Do jackknife to measure variable importance’ were chosen; the remaining model values were set to default values. Then, we evaluated the percent contribution and permutation contribution of the environmental variables by the Jackknife test to select key environmental variables for modeling. Finally, the occurrence points and key environmental variables were uploaded to MaxEnt to simulate the distribution of *D. citri* in China. In the final model, “Random seed” was chosen, and 10 replicate models were run. We selected the best model with the highest AUC value. The remaining model settings were set to the same as the initial model^39,50,59,60^.

The file output by the MaxEnt model is in ASCII format, and it cannot be visually displayed on the map. “Conversion Tools” in ArcGIS was used to convert the file from ‘ASCII’ to ‘Raster’ format, and the “Extraction” function was used to extract the probability distribution map of *D. citri* in China. We reclassified the distribution threshold and divided the suitable area into 4 categories, displaying them in different colors according to Wang’s method^50,61^. The specific description is shown in Table 1.

**Table 1:** Standards of the probability in this research^50^.

Habitat type	Standards	Color
Unsuitable area	P ≤ 0.05	White
Marginally suitable area	0.05 < P ≤ 0.33	Yellow
Moderately Suitable area	0.33 < P ≤ 0.66	Orange
Most suitable area	P > 0.66	Red

The receiver operating characteristic curve (ROC) is an effective method for use when evaluating the accuracy of the species distribution model. ROC curves are widely used in the evaluation of species distribution models^39,62^. The method sets the area under the curve (AUC) as the index to measure the accuracy. The theoretical value range of AUC is 0.5~1; the closer the AUC value is to 1, the higher the prediction accuracy of the model. The evaluation criteria are simulation failure (fail), 0.5 ≤ AUC < 0.6; poor simulation results (poor), 0.6 ≤ AUC < 0.7; generally fair simulation results (fair), 0.7 ≤ AUC < 0.8; good simulation results (good), 0.8 ≤ AUC < 0.9; and excellent simulation results (excellent), 0.9 ≤ AUC < 1^50^.

Arcgis10.0 software was used for map processing (Figs. 4, 8).

## Supplementary information

Supplementary Table S1.

Supplementary Table S2.

## Data Availability

Occurrence data of *D. citri* are available from Figshare at: 10.6084/m9.figshare.7564685.
