# Supplementary material for: Predictions of potential geographical distribution of Diaphorina citri (Kuwayama) in China under climate change scenarios
Source: Sci Rep. 2020 Jun 8;10:9202. doi: 10.1038/s41598-020-66274-5 (PMC7280263; doi:10.1038/s41598-020-66274-5)
Supplement: Supplementary file 1 — Supplementary Table S1. [file 41598_2020_66274_MOESM1_ESM.pdf]

Predictions of potential geographical distribution of *Diaphorina citri* (Kuwayama) in China under climate change scenarios

Rulin Wang[1, 2], Hua Yang[3], Mingtian Wang[4], Zhe Zhang[1], Tingting Huang[2], Gang Wen[5], Qing Li[1,*]

[1]College of Agronomy, Sichuan Agricultural University, Chengdu, Sichuan, 611130, China. [2]Sichuan Provincial Rural Economic Information Center, Chengdu, Sichuan, 610072, China. [3]Key Laboratory of Ecological Forestry Engineering of Sichuan Province/College of Forestry, Sichuan Agricultural University, Chengdu 611130, China. [4]Sichuan Meteorological Observatory, Chengdu, Sichuan, 610072, China. [5] *Bureau of Agriculture of Yibin City,* Yibin, Sichuan, 644000, China. Correspondence and requests for materials should be addressed to Q.L. (email: liq8633@163.com)

S1 Table. List of environmental variables used for this study, with type and measurement unit

Code	Environmental variables	Unit
bio1	Annual Mean Temperature	℃
bio2	Mean Diurnal Range (Mean of monthly (max temp - min temp)	℃
bio3	Isothermality (bio2/bio7) (* 100)	-
bio4	Temperature Seasonality (standard deviation *100)	-
bio5	Max Temperature of Warmest Month	℃
bio6	Min Temperature of Coldest Month	℃
bio7	Temperature Annual Range (bio5-bio6)	℃
bio8	Mean Temperature of Wettest Quarter	℃
bio9	Mean Temperature of Driest Quarter	℃
bio10	Mean Temperature of Warmest Quarter	℃
bio11	Mean Temperature of Coldest Quarter	℃
bio12	Annual Precipitation	mm
bio13	Precipitation of Wettest Month	mm
bio14	Precipitation of Driest Month	mm
bio15	Precipitation Seasonality (Coefficient of Variation)	mm
bio16	Precipitation of Wettest Quarter	mm
bio17	Precipitation of Driest Quarter	mm
bio18	Precipitation of Warmest Quarter	mm
bio19	Precipitation of Coldest Quarter	mm
