# Supplementary material for: Predictions of potential geographical distribution of Diaphorina citri (Kuwayama) in China under climate change scenarios
Source: Sci Rep. 2020 Jun 8;10:9202. doi: 10.1038/s41598-020-66274-5 (PMC7280263; doi:10.1038/s41598-020-66274-5)
Supplement: Supplementary file 2 — Supplementary Table S2. [file 41598_2020_66274_MOESM2_ESM.pdf]

Predictions of potential geographical distribution of *Diaphorina citri* (Kuwayama) in China under climate change scenarios

Rulin Wang[1, 2], Hua Yang[3], Mingtian Wang[4], Zhe Zhang[1], Tingting Huang[2], Gang Wen[5], Qing Li[1,*]

[1]College of Agronomy, Sichuan Agricultural University, Chengdu, Sichuan, 611130, China. [2]Sichuan Provincial Rural Economic Information Center, Chengdu, Sichuan, 610072, China. [3]Key Laboratory of Ecological Forestry Engineering of Sichuan Province/College of Forestry, Sichuan Agricultural University, Chengdu 611130, China. [4]Sichuan Meteorological Observatory, Chengdu, Sichuan, 610072, China. [5] *Bureau of Agriculture of Yibin City,* Yibin, Sichuan, 644000, China. Correspondence and requests for materials should be addressed to Q.L. (email: liq8633@163.com)

S2 Table. Pairwise Pearson's correlation coefficients of environmental variables.

	alt	bio1	bio2	bio3	bio4	bio5	bio6	bio7	bio8	bio9	bio10	bio11	bio12	bio13	bio14	bio15	bio16	bio17	bio18
**bio1**	0.1																		
**bio2**	0.15	0.37**																	
**bio3**	-0.05	0.22*	0.24**																
**bio4**	0.5**	0.62**	0.41**	0.13															
**bio5**	0.28**	0.57**	0.44**	0.13	0.57**														
**bio6**	-0.11	0.44**	0.15	0.06	0.4**	0.5**													
**bio7**	0.25**	0.81**	0.36**	0.14	0.68**	0.61**	0.46**												
**bio8**	0.15	0.91**	0.22*	-0.08	0.49**	0.64**	0.41**	0.71**											
**bio9**	-0.03	0.62**	0.21*	0.02	0.59**	0.41**	0.72**	0.58**	0.55**										
**bio10**	0.3**	0.59**	0.53**	0.16	0.69**	0.52**	0.22**	0.58**	0.53**	0.46**									
**bio11**	-0.07	0.56**	0.2*	0.01	0.49**	0.36**	0.75**	0.55**	0.54**	0.8**	0.44**								
**bio12**	0.33**	0.53**	0.37**	0.13	0.77**	0.48**	0.24**	0.59**	0.6**	0.49**	0.59**	0.33**							
**bio13**	0.13	0.3**	0.17*	-0.07	0.59**	0.3**	0.39**	0.29**	0.29**	0.49**	0.41**	0.57**	0.38**						
**bio14**	-0.11	0.78**	0.31**	0.05	0.36**	0.53**	0.55**	0.7**	0.81**	0.72**	0.48**	0.59**	0.37**	0.12					
**bio15**	-0.01	0.1	0.73**	0.14	0.19*	0.31**	0.14	0.06	0.05	0.09	0.39**	0.07	0.21*	0.14	0.18*				
**bio16**	0.71**	0.28**	0.17*	-0.08	0.7**	0.39**	0.05	0.43**	0.44**	0.23*	0.45**	0.1	0.51**	0.33**	0.1	0.02			
**bio17**	0.38**	0.61**	0.3**	0.01	0.72**	0.57**	0.6**	0.68**	0.6**	0.79**	0.59**	0.62**	0.62**	0.4**	0.69**	0.14	0.55**		
**bio18**	**0.92****	0.22*	0.23**	-0.01	0.62**	0.39**	0.02	0.35**	0.31**	0.11	0.42**	0.08	0.42**	0.27**	0	0.07	0.74**	0.5**	
**bio19**	0.33**	0.66**	0.32**	0.03	0.75**	0.56**	0.49**	0.73**	0.59**	0.71**	0.6**	0.69**	0.63**	0.4**	0.66**	0.1	0.51**	0.79**	.44**

Notes:

bio1: Annual Mean Temperature; bio2: Mean Diurnal Range; bio3: Isothermality; bio4: Temperature Seasonality; bio5: Max Temperature of Warmest Month; bio6: Min Temperature of Coldest Month; bio7: Temperature Annual Range; bio8: Mean Temperature of Wettest Quarter; bio9: Mean Temperature of Driest Quarter; bio10: Mean Temperature of Warmest Quarter; bio11: Mean Temperature of Coldest Quarter; bio12: Annual Precipitation; bio13: Precipitation of Wettest Month; bio14:

Precipitation of Driest Month; bio15: Precipitation Seasonality; bio16: Precipitation of Wettest Quarter; bio17: Precipitation of Driest Quarter; bio18: Precipitation of Warmest Quarter; bio19: Precipitation of Coldest Quarter; ALT: Altitude. The symbol '**' indicates a significant correlation at the level of alpha = 0.01.
